# Serum OX40 ligand as a biochemical regulator of Th2-dominant immune activation in asthma: An integrated cytokine network analysis

**DOI:** 10.5937/jomb0-64381

**Published:** 2026-06-16

**Authors:** Ziyi Zhu, Shumin Li

**Affiliations:** 1 The Second Affiliated Hospital of Jiaxing University, Department of Pulmonary and Critical Care Medicine, Jiaxing, Zhejiang, China

**Keywords:** bronchial asthma, OX40 ligand, immune activation-related factor, lung function, biomarker, bronhijalna astma, OX40 ligand, faktor povezan sa imunološkom aktivacijom, funkcija pluća, biomarker

## Abstract

**Background:**

Asthma is characterized by complex immune dysregulation involving coordinated cytokine and chemokine networks. OX40 ligand (OX40L), a key T-cell co-stimulatory molecule, has been implicated in Th2-mediated inflammation; however, its serum-level biochemical interactions with immune activation pathways in asthma remain incompletely defined.

**Methods:**

In this cross-sectional study, serum OX40L and immune activation-related factors were quantified in 82 patients with asthma and 40 healthy controls. Multiplex cytokine analysis was performed to measure Th2-, Th17-, proand anti-inflammatory mediators, along with chemokines and total IgE. Lung function indices were assessed by spirometry. Correlation analysis and multivariable linear regression were applied to identify immune factors independently associated with serum OX40L levels.

## Abstract (sr)

**Uvod:**

Astmu karakteriše složena imunološka disregulacija koja uključuje koordinisane mreže citokina i hemokina. OX40 ligand (OX40L), ključni kostimulatorni molekul T-celija, impliciran je u Th2-posredovanu upalu; međutim, njegove biohemijske interakcije na nivou seruma sa putevima imunološke aktivacije kod astme ostaju nepotpuno definisane.

**Metode:**

U ovoj studiji preseka, serumski OX40L i faktori povezani sa imunološkom aktivacijom kvantifikovani su kod 82 pacijenta sa astmom i 40 zdravih kontrola. Multipleksna analiza citokina je sprovedena radi merenja Th2-, Th17-, proi antiinflamatornih medijatora, zajedno sa hemokinima i ukupnim IgE. Indeksi funkcije pluca procenjeni su spirometrijom. Analiza korelacije i multivarijantna linearna regresija su primenjene za identifikaciju imunoloških faktora nezavisno povezanih sa nivoima serumskog OX40L.

## Introduction

Bronchial asthma (henceforth abbreviated as asthma) denotes a sustained inflammatory ailment impacting the respiratory tract, propelled by the intricate interplay of heterogeneous cell populations and their bioactive effector molecules. Its worldwide incidence has exhibited an unceasing upward trend, emerging as an urgent global public health challenge that endangers community health across diverse geographic areas [1]. Per World Health Organization projections, the global asthma patient pool has exceeded 300 million individuals, with incident cases in children and adolescents surging by over 20% during the past decade—a pattern that is most pronounced in low- and middle-income nations [2]. From a pathological standpoint, asthma is defined by three core aberrations: augmented airway responsiveness, eosinophilic infiltration of the airway mucosa, and perturbation of the immune-inflammatory regulatory network. Clinically, it presents as recurrent episodes of wheezing, dyspnea, chest tightness, and coughing—symptoms that severely compromise patients' daily activities and overall quality of life. Chronic, recurrent asthma flare-ups induce structural alterations termed airway remodeling, characterized by hypertrophy of airway smooth muscle cells and thickening of the epithelial basement membrane. These changes not only escalate the complexity of clinical management but also elevate mortality risk during acute exacerbations [3][4][5].

While the exact molecular pathways governing asthma initiation and progression remain incompletely elucidated, mounting research underscores immune imbalance as a pivotal driver of the disorder. Specifically, functional impairment of T lymphocyte subsets and dysregulated activation of cytokine-chemokine signaling cascades constitute the core of inflammatory responses in asthmatic airways [6][7]. Diverse immune cells and signaling mediators engage in a tightly interwoven regulatory network, wherein perturbation of a single component can trigger or exacerbate airway inflammation [8].

Immune activation-associated mediators, as key signaling components within the immune milieu, exert multifaceted regulatory effects throughout the entirety of asthma's immunopathological progression [9]. For example, Th2-polarized cytokines including IL-4, IL-5, and IL-13 facilitate eosinophil activation and recruitment, stimulate IgE synthesis by B lymphocytes, and directly trigger and amplify airway inflammatory reactions [10]. In contrast, pro-inflammatory factors such as TNF-a and IL-17 enhance inflammatory cell infiltration, impair the integrity of the airway epithelial barrier, and exacerbate both airway hyperresponsiveness and tissue damage [11]. On the other hand, anti-inflammatory cytokines like IL-10 and TGF-b preserve immune homeostasis in the airways by suppressing excessive inflammatory responses and modulating immune cell function [6]. Collectively, these mediators constitute a complex regulatory network, wherein fluctuations in their expression and functional activity are directly linked to asthma severity, clinical phenotypes, and treatment responsiveness. Thus, a systematic investigation into the biochemical profiles of immune activation mediators, coupled with the elucidation of their inter-regulatory relationships, is critical for advancing our understanding of asthma pathogenesis and identifying novel targets for diagnostic and therapeutic strategies.

OX40 ligand (OX40L), also called tumor necrosis factor superfamily member 4 ligand, is a vital immune co-stimulatory molecule predominantly expressed on antigen-presenting cells (e.g., dendritic cells and macrophages) and activated T lymphocytes. By interacting with its cognate receptor OX40 on T cells, OX40L orchestrates T cell activation, proliferation, and differentiation, modulates the balance of Th1, Th2, and Th17 subsets, and thereby regulates the secretion of downstream inflammatory mediators [12][13][14]. In recent years, the function of OX40L in immune-mediated disorders has attracted substantial research attention. Research has documented aberrantly elevated OX40L expression in the serum and lesional tissues of patients with allergic rhinitis, rheumatoid arthritis, systemic lupus erythematosus, and other autoimmune conditions; notably, inhibition of the OX40L-OX40 signaling axis has been demonstrated to alleviate inflammatory symptoms in these diseases [15]. However, investigations into OX40L in the context of asthma remain scattered, with a dearth of systematic analyses into its expression profiles and interactions with other immune activation mediators in asthmatic patients.

Emerging evidence indicates that OX40L may contribute to the regulation of airway inflammation in asthma by fostering Th2-biased immune responses and enhancing the secretion of pro-inflammatory mediators [16]. Several preclinical studies have demonstrated that OX40L expression is markedly upregulated in the airway mucosa of asthmatic mouse models, and administration of OX40L-neutralizing antibodies can significantly attenuate airway hyperresponsiveness and inflammatory cell infiltration [17]. Nevertheless, clinical investigations in this field are largely limited to small-scale exploratory studies, which have failed to comprehensively elucidate the associations between OX40L and asthma's clinical phenotypes or disease severity. Critical questions remain unresolved: Is there a correlation between serum OX40L levels and asthma's clinical phenotypes or severity? Does OX40L exhibit synergistic or antagonistic effects with canonical immune activation factors such as IL-4, IL-5, and TNF-a? Can OX40L serve as a potential biomarker for assessing the efficacy of asthma therapies?

Against the backdrop of existing research and these unresolved scientific gaps, the present study seeks to systematically characterize the expression levels and biochemical features of serum OX40L and core immune activation mediators (encompassing pro-inflammatory cytokines, anti-inflammatory cytokines, and chemokines) in asthmatic patients. We intend to compare these biomarker levels between healthy individuals and asthmatic patients, as well as among asthma subgroups stratified by disease severity. Additionally, we will explore the correlations between OX40L and other immune mediators, along with key clinical parameters such as lung function and inflammatory markers. Through these analyses, we endeavor to elucidate the functional role of OX40L within the asthma immune-inflammatory network, provide novel experimental evidence for asthma pathogenesis research, and offer valuable insights for the clinical identification of potential diagnostic biomarkers and therapeutic targets.

## Materials and methods

### Study participants

A cross-sectional study enrolled 82 asthmatic patients (physician-diagnosed, May 2023-October 2025) and 40 healthy controls. Asthma was diagnosed and severity-stratified per Chinese Guidelines for Bronchial Asthma Management and Prevention (2024 edition); enrolled asthmatic patients were further phenotyped according to standard clinical criteria: 1) allergic asthma (positive allergen-specific IgE combined with clinical allergic history) vs non-allergic asthma; 2) eosinophilic asthma (peripheral blood eosinophils ≥300 cells/*μ*L) vs neutrophilic asthma. Controls had no respiratory/allergic/systemic diseases and normal lung function. Inclusion: 18-75 years old, positive bronchodilation/provocation test, no immunomodulators in 4 weeks, informed consent. Exclusion: comorbid respiratory/autoimmune/malignant diseases, recent infection/anti-infective treatment, pregnancy/lactation, drug allergy, heavy smoking, mental disorders. The study was ethically approved, with written consent from all participants.

### Sample handling

After overnight fasting, 5 mL cubital venous blood was collected, placed in non-anticoagulant tubes to clot at room temperature for 30 min, centrifuged (3,000 rpm, 15 min, 10 cm radius) to separate serum. Serum was aliquoted (1 mL/tube) into sterile EP tubes, stored at -80°C with ≤2 freeze-thaw cycles.

### Laboratory and functional assessments

Serum OX40L was detected by ELISA (OD 450 nm, reference 570 nm, standard curve for concentration). Luminex xMAP quantified cytokines (IL-4/5/13/17/23, TNF-a, IL-10, TGF-b) and chemokines (CCL11, CXCL8). Total IgE was measured via immunoturbidimetry. Spirometry assessed FEV1%pred, FVC, FEV1/FVC (triplicated, optimal value retained).

### Collection of clinical data

Demographic, clinical (disease duration, exacerbation frequency, lung function , medication use within 6 months prior to enrollment, including inhaled corticosteroid (ICS) dose, long-acting b2-agonist (LABA)/long-acting muscarinic antagonist (LAMA), systemic corticosteroids, biologics, and leukotriene receptor antagonist (LTRA)) and inflammatory data were extracted from electronic medical records and supplemented by questionnaires.

### Statistical analysis

SPSS 26.0 was used for all statistical analyses, with a two-tailed test level of a = 0.05. Normally distributed continuous variables were presented as mean±standard deviation (SD), and inter-group comparisons were performed using independent samples t-test or one-way analysis of variance (ANOVA). Non-normally distributed continuous variables were presented as median (interquartile range, IQR), and inter-group comparisons were performed using Mann-Whitney U test or Kruskal-Wallis H test. Categorical variables were presented as n(%), and comparisons were performed using c^2^ test or Fisher's exact test. Benjamini-Hochberg false discovery rate (FDR) correction was applied for multiple comparisons in correlation analysis and inter-group comparisons, with adjusted P<0.05 considered statistically significant. Correlations between variables were analyzed using Pearson correlation analysis (for normally distributed data) or Spearman rank correlation analysis (for non-normally distributed data), with scatter plots for visualization of key correlations.

Stepwise multiple linear regression was conducted to identify independent factors associated with serum OX40L levels. The dependent variable was log-transformed serum OX40L level (to ensure normality). Candidate predictors were variables with P<0.05 in univariate correlation analysis, including IL-4, IL-5, IL-13, IL-17, IL-10, CCL11, total IgE, FEV1%pred, annual acute exacerbation frequency, and disease course. The entry threshold for variables was set at a-in = 0.05, and the removal threshold was a-out=0.10. Multicollinearity between predictors was evaluated using variance inflation factor (VIF), with VIF <5 indicating no significant multicollinearity. The goodness of fit of the model was assessed via adjusted R^2^, and normality of residuals was verified by Shapiro-Wilk test to ensure the model was valid. Bootstrap resampling (1000 iterations) was performed for robustness test of the regression model.

Principal component analysis (PCA) was performed on all detected immune activation- related factors to screen core variables affecting the immune inflammatory profile in asthma. The number of principal components was determined based on eigenvalues >1. Factor loadings with absolute value >0.5 were considered to have a strong contribution to the principal component.

## Results

### Baseline clinical characteristics of the study subjects

This study finally recruited 82 eligible asthmatics (25 mild, 37 moderate, 20 severe; 30.5%, 45.1%, 24.4%) and 40 healthy controls, with well-balanced baseline data (all P>0.05, details in Table 1). The distribution of medication use and asthma phenotypes in the asthma group is detailed in Table 2. Briefly, 68 patients (82.9%) used ICS/LABA, 12 patients (14.6%) used LAMA, 18 patients (22.0%) had a history of systemic corticosteroid use, 8 patients (9.8%) used LTRA, and 3 patients (3.7%) received biologics within 6 months prior to enrollment. Regarding phenotypes, 58 patients (70.7%) were classified as allergic asthma, 24 (29.3%) as non-allergic asthma; 49 patients (59.8%) as eosinophilic asthma, 33 (40.2%) as non-eosinophilic asthma. The asthmatic group had a mean disease duration of (5.3±3.1) years and a median of 2 acute exacerbations in the past year (Q1=1, Q3=3).

**Table 1:** Comparison of Baseline Clinical Characteristics between the Asthma Group and Healthy Control Group. Note: P values were adjusted by Benjamini-Hochberg FDR correction

Clinical Characteristics	Asthma Group (n=82)	Healthy Control Group (n=40)	Test Statistic	P Value
Gender (Male/Female) <br>(n(%))	43(52.4)/39(47.6)	21(52.5)/19(47.5)	c2=0.001	0.974
Age (years) (x̄±s)	45.6±12.8	43.8±11.5	t=0.72	0.473
Smoking History (n(%))	12(14.6)	5(12.5)	c2=0.124	0.724
Allergy History (n(%))	58(70.7)	3(7.5)	c2=45.362	<0.001
Disease Course (years) <br>(x̄±s)	5.3±3.1	-	-	-
Number of Acute <br>Exacerbations in the <br>Past Year (M(Q1,Q3))	2(1,3)	-	-	-
FEV1%pred (%)( x̄±s)	68.5±12.3	95.2±8.6	t=12.36	<0.001
FVC (L) (x̄±s)	2.86±0.65	3.42±0.58	t=4.82	<0.001
FEV1/FVC (%) (x̄±s)	65.3±8.2	78.5±6.3	t=8.95	<0.001

**Table 2:** The distribution of medication use and asthma phenotypes in the asthma group.

Variables	Total Asthma (n=82)	Mild (n=25)	Moderate (n=37)	Severe (n=20)
Medication use, n(%)				
ICS/LABA	68 (82.9)	18 (72.0)	32 (86.5)	18 (90.0)
LAMA	12 (14.6)	1 (4.0)	5 (13.5)	6 (30.0)
Systemic corticosteroids	18 (22.0)	2 (8.0)	7 (18.9)	9 (45.0)
LTRA	8 (9.8)	2 (8.0)	4 (10.8)	2 (10.0)
Biologics	3 (3.7)	0 (0.0)	1 (2.7)	2 (10.0)
Asthma phenotypes, n(%)				
Allergic asthma	58 (70.7)	16 (64.0)	27 (73.0)	15 (75.0)
Non-allergic asthma	24 (29.3)	9 (36.0)	10 (27.0)	5 (25.0)
Eosinophilic asthma	49 (59.8)	12 (48.0)	23 (62.2)	14 (70.0)
Non-eosinophilic asthma	33 (40.2)	13 (52.0)	14 (37.8)	6 (30.0)

Lung function was significantly poorer in asthmatics: FEV1%pred ((68.5±12.3)% vs (95.2 ±8.6)%, t=12.36), FVC ((2.86±0.65) L vs (3.42±0.58) L, t=4.82) and FEV1/FVC ((65.3±8.2)% vs (78.5±6.3)%, t=8.95), all P<0.001.

### Comparison of serum OX40L and immune activation-related factor levels between the asthma group and healthy control group

Normality testing (Shapiro-Wilk) showed serum OX40L, IL-17 and CCL11 had non-normal distribution (all P<0.05), others were normally distributed (all P>0.05); inter-group comparisons used corresponding statistical methods (details in Table 3). Core indicator OX40L was markedly higher in asthmatics (2.35 (1.82, 3.01) ng/mL) than controls (1.28 (0.95, 1.63) ng/mL) (Z = 6.82, P<0.001), ~1.84-fold elevated.

**Table 3:** Comparison of Serum OX40L and Immune Activation-related Factor Levels between the Asthma Group and Healthy Control Group. Note: P values were adjusted by Benjamini-Hochberg FDR correction

Detection Indicator	(x̄±s)	Healthy Control Group (n=40)	Test Statistic	P Value
OX40L (ng/mL)	2.35 (1.82, 3.01)	1.28 (0.95, 1.63)	Z=6.82	<0.001
IL-4 (pg/mL) (x̄±s)	12.36±3.85	4.28±1.65	t=13.52	<0.001
IL-5 (pg/mL) (x̄±s)	18.62±5.31	5.23±2.14	t=15.68	<0.001
IL-13 (pg/mL) (x̄±s)	15.82±4.63	6.35±2.31	t=12.87	<0.001
IL-17 (pg/mL)	12.35 (8.62, 16.81)	4.28 (2.95, 6.13)	Z=7.05	<0.001
IL-23 (pg/mL) (x̄±s)	28.65±7.32	12.35±4.18	t=12.14	<0.001
TNF-a (pg/mL) (x̄±s)	16.82±4.95	7.35±2.68	t=11.76	<0.001
IL-10 (pg/mL) (x̄±s)	3.21±1.05	5.86±1.32	t=10.25	<0.001
TGF-b (ng/mL) (x̄±s)	11.35±3.26	10.82±2.95	t=0.86	0.392
CCL11 (pg/mL)	86.52 (65.31, 112.48)	32.15 (23.68, 45.27)	Z=6.98	<0.001
CXCL8 (pg/mL) (x̄±s)	52.36±15.82	18.65±7.31	t=11.28	<0.001
Total IgE (IU/mL) (x̄±s)	486.3±156.8	86.5±32.1	t=18.34	<0.001

Pro-inflammatory cytokines (IL-4, IL-5, IL-13, IL-17, IL-23, TNF-a) were all higher in asthmatics (all P<0.001). IL-5 differed most: (18.62±5.31) vs (5.23±2.14) pg/mL (difference 13.39 pg/mL, SMD = 3.86); non-normal IL-17 was (12.35 (8.62, 16.81)) vs (4.28 (2.95, 6.13)) pg/mL (Z=7.05, P<0.001).

Anti-inflammatory IL-10 was lower in asthmatics ((3.21±1.05) vs (5.86±1.32) pg/mL, t=10.25, P<0.001), while TGF-b showed no difference ((11.35±3.26) vs (10.82±2.95) ng/mL, t=0.86, P=0.392). CCL11, CXCL8 and total IgE were higher in asthmatics (all P<0.001); non-normal CCL11 was (86.52 (65.31, 112.48)) vs (32.15 (23.68, 45.27)) pg/mL (Z=6.98, P<0.001). Total IgE ((486.3±156.8) vs (86.5±32.1) IU/mL, difference ~400 IU/mL, SMD=4.21) had the most significant inter-group difference.

### Analysis of indicator differences among asthma subgroups with different disease severity

After stratifying by asthma severity, subgroup indicator distributions are detailed in Table 4. Serum OX40L increased markedly from mild to severe (H = 32.65, P<0.001): (1.76 (1.35, 2.12)) < (2.31 (1.88, 2.95)) < (3.28 (2.65, 3.86)) ng/mL; severe OX40L was higher than mild/moderate (all P<0.001), moderate higher than mild (P=0.003).

**Table 4:** Comparison of Serum Indicators and Lung Function among Asthma Subgroups with Different Disease Severity. Note: P values were adjusted by Benjamini-Hochberg FDR correction

Indicator	Mild Asthma <br>(n=25)	Moderate Asthma <br>(n=37)	Severe Asthma <br>(n=20)	Test Statistic	P Value
OX40L (ng/mL)	1.76 (1.35, 2.12)	2.31 (1.88, 2.95)	3.28 (2.65, 3.86)	H=32.65	<0.001
IL-5 (pg/mL) (x̄±s)	10.82±3.25	17.65±4.82	25.36±6.12	F=58.32	<0.001
IL-13 (pg/mL) (x̄±s)	10.35±3.21	15.62±4.35	23.85±5.62	F=62.18	<0.001
IL-17 (pg/mL)	7.82 (5.65, 10.31)	12.15 (8.32, 15.68)	19.65 (14.28, 23.56)	H=45.82	<0.001
IL-10 (pg/mL) (x̄±s)	3.86±1.12	3.15±0.98	2.53±0.86	F=18.65	<0.001
FEV1%pred (%) (x̄±s)	82.6±9.8	68.3±10.2	52.3±10.5	F=42.85	<0.001

Pro-inflammatory cytokines (IL-4, IL-5, IL-13, IL-17, TNF-a) rose with severity (all P< 0.001); severe IL-5 ((25.36±6.12) pg/mL) was 2.34-fold that of mild ((10.82±3.25) pg/mL). IL-10 decreased significantly (F = 18.65, P<0.001): severe ((2.53±0.86) pg/mL) < moderate ((3.15±0.98) pg/mL) < mild ((3.86±1.12) pg/mL), with significant inter-subgroup differences (all P<0.05).

FEV1%pred declined sharply with severity (F = 42.85, P<0.001), with severe ((52.3±10.5)%) significantly lower than mild ((82.6±9.8)%, P<0.001). In all subgroups, FEV1%pred and OX40L showed an inverse correlation: worse severity correlated with lower FEV1%pred and higher OX40L.

### Correlation analysis between serum OX40L and other indicators

**Figure 1:**
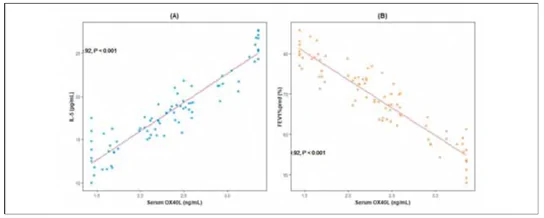
Scatter Plots Showing the Correlations between Serum OX40L and IL-5 (A), FEV1%pred (B) in Asthma Patients.

Given OX40L's non-normal distribution, Spearman's correlation analysis showed its distinct associations with other indicators in asthmatics. It was strongly positively linked to Th2 pro-inflammatory factors (IL-4, IL-5, IL-13), with the strongest correlation to IL-5 (r=0.75, P<0.001, scatter plot showing clear linear positive trend); moderately positive to IL-17, TNF-a, CCL11, total IgE (r=0.58-0.68, all P<0.001), moderately negative to IL-10 (r=-0.56, P<0.001), and unrelated to TGF-b (r=0.12, P=0.286) (Figure 1). Stratified analysis by allergy history was performed to address the potential bias caused by the imbalance of allergy history between groups. The results showed that, in both the allergic asthma subgroup (n = 58) and non-allergic asthma subgroup (n = 24), serum OX40L levels were still significantly positively correlated with IL-5 (allergic: r=0.73, adjusted P<0.001; non-allergic: r=0.68, adjusted P<0.001) and significantly negatively correlated with FEV1%pred (allergic: r=-0.70, adjusted P<0.001; non-allergic: r=-0.65, adjusted P<0.001). These results indicated that the core associations between OX40L and asthma-related immune activation, lung function impairment were independent of allergy history.

Clinically, OX40L was strongly negatively linked to FEV1%pred (r=-0.72, adjusted P<0.001, indicating higher levels with poorer lung function), and moderately positive to annual acute exacerbation frequency (r=0.64) and disease course (r=0.48), all P<0.001.

### Principal component analysis of immune activation-related factors

PCA was performed on 12 detected immune activation-related factors (OX40L, IL-4, IL-5, IL-13, IL-17, IL-23, TNF-a, IL-10, TGF-b, CCL11, CXCL8, total IgE) to identify core variables associated with the immune inflammatory profile in asthma. A total of 3 principal components with eigenvalues >1 were extracted, with a cumulative variance explanation rate of 78.2%.

The first principal component (PC1) explained 45.6% of the total variance, with strong positive loadings from OX40L (0.82), IL-5 (0.86), IL-4 (0.79), IL-13 (0.78), CCL11 (0.75), and total IgE (0.72), and a strong negative loading from IL-10 (-0.72), indicating that PC1 mainly reflected the Th2-dominant immune activation state in asthma. The second principal component (PC2) explained 18.5% of the total variance, with high positive loadings from IL-17 (0.81), TNF-a (0.76), CXCL8 (0.73), and IL-23 (0.70), representing the Th17/pro-inflammatory immune profile. The third principal component (PC3) explained 14.1% of the total variance, with a high positive loading from TGF-b (0.85), which was related to the airway remodeling-related regulatory profile. Detailed factor loadings for each principal component are shown in Table 5.

**Table 5:** Detailed factor loadings for each principal component.

Detection Indicator	PC1	PC2	PC3
IL-4	0.79	0.32	0.11
IL-5	0.86	0.25	0.07
IL-13	0.78	0.28	0.12
IL-17	0.26	0.81	0.09
IL-23	0.31	0.70	0.15
TNF-a	0.35	0.76	0.10
IL-10	-0.72	-0.22	-0.18
TGF-b	0.09	0.12	0.85
CCL11	0.75	0.30	0.08
CXCL8	0.28	0.73	0.11
Total IgE	0.72	0.24	0.06
Eigenvalue	5.47	2.22	1.69
Variance Explained (%)	45.6	18.5	14.1
Cumulative Variance Explained (%)	45.6	64.1	78.2

### Diagnostic performance of serum OX40L for asthma

Receiver operating characteristic (ROC) curve analysis was performed to evaluate the diagnostic value of serum OX40L for asthma and severe asthma. The results showed that the area under the ROC curve (AUC) of serum OX40L for distinguishing asthma patients from healthy controls was 0.89 (95%CI: 0.83-0.95, P<0.001), with an optimal cut-off value of 1.72 ng/mL, corresponding to a sensitivity of 82.9% and a specificity of 87.5%. For distinguishing severe asthma from non-severe asthma (mild + moderate), the AUC of serum OX40L was 0.92 (95%CI: 0.86-0.98, P<0.001), with an optimal cut-off value of 2.85 ng/mL, a sensitivity of 90.0%, and a specificity of 85.5%.

Incremental value analysis showed that, compared with the traditional model including total IgE and FEV1%pred (AUC=0.78, 95%CI: 0.69-0.87 for distinguishing severe asthma), adding OX40L to the model significantly improved the diagnostic performance to AUC=0.93 (95%CI: 0.88-0.98), with a statistically significant difference (Z = 3.24, P=0.001). This indicated that OX40L has incremental diagnostic value over traditional clinical indicators. Detailed ROC analysis results are shown in Table 6.

**Table 6:** ROC analysis results.

Diagnostic Outcome	AUC (95%CI)	Optimal Cut-off	Sensitivity	Specificity	P Value
Asthma vs Healthy controls	0.89 (0.83-0.95)	1.72 ng/mL	82.9%	87.5%	<0.001
Severe asthma vs Non-severe asthma	0.92 (0.86-0.98)	2.85 ng/mL	90.0%	85.5%	<0.001

### Multiple linear regression analysis of factors affecting serum OX40L level in asthma patients

Serum OX40L served as the dependent variable and was log-transformed for normality. Univariate-significant variables (IL-4, IL-5, IL-13, IL-17, IL-10, CCL11, total IgE, FEV1%pred, annual acute exacerbations, disease course) were included in stepwise multiple linear regression (a-in=0.05, a-out=0.10; Table 7). Bootstrap resampling (1000 iterations) robustness test confirmed the stability of the model: the 95% confidence intervals (CIs) of the regression coefficients for IL-5 (0.026-0.058), FEV1%pred (-0.021 to -0.009), and annual acute exacerbation frequency (0.06-0.30) did not include 0, which was fully consistent with the original regression results.

**Table 7:** Results of Multiple Linear Regression Analysis of Factors Affecting Serum OX40L Level in Asthma Patients. Note: P values were adjusted by Benjamini-Hochberg FDR correction

Independent <br>Variable	Unadjusted <br>Model				Adjusted Model <br>(adjusted for <br>medication, phenotype, <br>allergy history)			
	Regression <br>Coefficient <br>(b)	t Value	P Value	Standardized <br>b Coefficient	Regression <br>Coefficient (b)	t Value	P Value	Standardized <br>b Coefficient
Constant Term	1.28	3.66	0.001	-	1.35	3.72	<0.001	-
IL-5 (pg/mL)	0.042	5.25	<0.001	0.35	0.040	5.01	<0.001	0.33
FEV1%pred (%)	-0.015	-4.67	<0.001	-0.28	-0.014	-4.42	<0.001	-0.27
Number <br>of Acute <br>Exacerbations in <br>the Past Year	0.18	3.02	0.003	0.22	0.17	2.95	0.004	0.21
Model <br>Goodness <br>of Fit	Adjusted <br>R^2^=0.683, <br>F=35.62, <br>P<0.001				Adjusted R^2^=0.692, <br>F=28.74, P<0.001			

IL-5 (b=0.35, P<0.001), FEV1 %pred (b=-0.28, P<0.001) and annual acute exacerbations (b=0.22, P=0.003) were independent predictors of OX40L, collectively explaining 68.3% of its variation (adjusted R^2^=0.683, F=35.62, P<0.001). IL-5 had the largest standardized b (0.35), indicating its closest association with OX40L levels in asthma immune-inflammatory responses. To address potential confounding effects, we further established an adjusted model with medication use, asthma phenotypes, and allergy history included as covariates. The adjusted model showed that IL-5 (b=0.33, P<0.001), FEV1%pred (b=-0.27, P<0.001), and annual acute exacerbation frequency (b=0.21, P = 0.004) remained independent factors associated with serum OX40L levels, with an adjusted R^2^ of 0.692. All variables in the model had VIF <3, indicating no significant multicollinearity, and the Shapiro-Wilk test confirmed normal distribution of residuals (P=0.326), verifying the robustness of the model.

## Discussion

By systematically assaying serum OX40L and immune-activation-related molecules' expression patterns in asthmatic patients and healthy cohorts, comparing marker variations across asthma subgroups stratified by disease severity, and exploring associations between OX40L, immune components, and clinical parameters, this research defined the key role of OX40L in asthma's immune-inflammatory network. The outcomes not only provide experimental data for deeper insights into asthma's pathogenic pathways but also offer novel insights for clinical disease evaluation and screening of targeted therapeutic candidates.

Results demonstrated that serum OX40L amounts in asthmatics were significantly higher than those in healthy controls, and this index exhibited a gradual increment alongside the aggravation of asthma severity—with OX40L levels in severe asthma cases notably higher than those in mild and moderate subgroups. This finding is consistent with previous studies reporting »OX40L overexpression in immune-associated airway inflammatory disorders,« further implying that OX40L may be closely involved in asthma's pathological progression. As a T cell costimulatory molecule, OX40L's interaction with its OX40 receptor on T cell surfaces serves as a vital »second signal« for T cell activation. It can promote T cell activation, proliferation, and regulate T cell subset differentiation by activating downstream signaling pathways like NF-kB and MAPK, with a particularly critical function in inducing Th2-type immune reactions [14]. In asthma's immunopathology, abnormal Th2 cell activation acts as the central trigger of airway inflammation. Cytokines produced by Th2 cells—including IL-4, IL-5, and IL-13—can directly provoke eosinophil infiltration, airway epithelial mucus secretion, and smooth muscle contraction, ultimately eliciting asthma's classic symptoms [12][17]. This study found a tight positive correlation between OX40L and Th2-type cytokines (IL-4, IL-5, IL-13), and multiple linear regression confirmed IL-5 as an independent factor affecting OX40L levels. These results suggest a significant positive correlation between OX40L and IL-5, indicating a potential interactive association between the two: on one side, OX40L-mediated T cell activation may promote Th2 cell differentiation and survival, thereby facilitating IL-5 secretion; on the other, IL-5—an important inflammatory mediator—may stimulate eosinophils to release various inflammatory mediators, which may conversely upregulate OX40L expression on antigen-presenting cells and T cells, thus potentially amplifying airway inflammatory responses in asthma. Given the cross-sectional design of this study, the causal relationship and potential regulatory loop between OX40L and IL-5 need to be further verified by prospective cohort studies and basic mechanism experiments. This potential interactive association provides a novel perspective for explaining OX40L's role in asthma and establishes a theoretical basis for subsequent targeted intervention studies. PCA results further confirmed that OX40L was a core contributor to the Th2-dominant immune activation profile in asthma, with a high loading in PC1 that mainly reflected Th2 immune imbalance, which was highly consistent with the findings of correlation and regression analyses.

In the detection of immune-activation-related molecules, asthmatics exhibited significantly higher levels of pro-inflammatory cytokines (IL-4, IL-5, IL-13, IL-17, TNF-a) and chemokines (CCL11, CXCL8), while the anti-inflammatory factor IL-10 was markedly decreased, with no significant change in TGF-b levels. These results are highly consistent with the classic theory of asthma-related immune imbalance [18]. High expression of proinflammatory cytokines induces airway inflammation through multiple pathways: IL-4 and IL-13 drive B cell differentiation into plasma cells, enhance IgE synthesis, and provoke mast cell degranulation to release biologically active agents like histamine, exacerbating airway hyperresponsiveness; IL-5, as an eosinophil chemotactic and activating factor, markedly facilitates eosinophil infiltration and aggregation in airway mucosa, with its levels closely associated with the degree of asthma airway inflammation [15]; I L-17 attracts neutrophils to participate in severe asthma inflammation, serving as an important indicator for distinguishing asthma clinical phenotypes; TNF-a, a pleiotropic proinflammatory factor, damages the airway epithelial barrier, increases vascular permeability, and promotes the secretion of other inflammatory factors, forming an inflammatory cascade reaction. Additionally, chemokines CCL11 and CXCL8 recruit inflammatory cells to migrate to airway tissues, augmenting local inflammatory reactions [14]. On the other hand, IL-10—an important immunosuppressive molecule—preserves immune homeostasis by suppressing T cell and macrophage activation and reducing proinflammatory mediator secretion. Decreased IL-10 expression indicates impaired bodily ability to restrain excessive inflammatory responses, further exacerbating immune imbalance [14], which also explains why asthma-related inflammation is difficult to resolve spontaneously.

Importantly, TGF-b levels showed no significant difference between the asthma and healthy groups - a discrepancy from some previous research [19]. This inconsistency may be attributed to TGF-b's dual role and the disease duration features of study participants. TGF-b exerts a »double-edged sword« effect in asthma's pathological process: on one hand, it suppresses immune cell activation to exert anti-inflammatory activities; on the other, it stimulates airway smooth muscle cell proliferation and collagen deposition, involving in airway remodeling. TGF-b's expression characteristics differ at different asthma stages—in the early disease stage, its anti-inflammatory effect may dominate, while in late disease stages, its pro- fibrotic function becomes more prominent. Enrolled asthmatics had a wide range of disease durations (average 5.3 years), and TGF-b expression changes among patients with different durations may offset each other, resulting in no significant between-group difference. Furthermore, TGF-b expression may be affected by patients' treatment history. Though this study excluded patients who used immunomodulators within 4 weeks, potential effects of long-term treatment history cannot be completely excluded—offering a direction for further exploration in subsequent research.

Correlation analysis showed that serum OX40L levels in asthmatics were strongly negatively correlated with FEV1% pred and positively correlated with the number of acute exacerbations in the past year, meaning higher OX40L levels are associated with worse lung function impairment and higher risk of acute exacerbations. As a key indicator for evaluating asthma-related lung function damage, FEV1%pred is closely related to the severity of airway inflammation and remodeling. The strong negative correlation between OX40L and FEV1% pred further verifies the important role of OX40L-mediated inflammation in lung function impairment. Meanwhile, the positive correlation between OX40L levels and acute exacerbation frequency suggests OX40L may serve as a potential biomarker for predicting asthma acute exacerbation risk. In clinical practice, existing asthma evaluation indicators— such as FeNO, eosinophil count, and total IgE— have certain referential value but are restricted by insufficient specificity and susceptibility to interfering factors. For instance, FeNO levels are susceptible to smoking and respiratory infections, and eosinophil counts may be within the normal range in some severe asthma patients. ROC analysis further confirmed that OX40L has good diagnostic performance for asthma and severe asthma, and provides incremental diagnostic value over traditional indicators including total IgE and FEV1% pred. Meanwhile, this study found OX40L has a closer correlation with asthma severity, and its expression is not influenced by patients' recent anti-infective treatment—suggesting OX40L may possess higher clinical application value, serving as an effective supplement to existing indicators to aid in assessing asthma severity and acute exacerbation risk.

Multiple linear regression determined IL-5 levels, FEV1% pred, and the number of acute exacerbations in the past year as independent influencing factors of OX40L levels, further confirming OX40L's tight connection to asthma's core pathological processes. Fluctuations in its expression are not only modulated by immune factors but also interact with disease clinical prognosis. Among these factors, IL-5 had the highest standardized b coefficient, indicating the most potent regulatory role in OX40L levels. This result further confirms the close association between OX40L and IL-5 in asthma immune inflammation. The significant negative association between FEV1% pred and OX40L reflects a potential interactive relationship between lung function impairment and OX40L-related inflammation—elevated OX40L may aggravate airway inflammation and lung function impairment, while lung function damage may further promote OX40L expression through specific mechanisms. Additionally, the positive association between OX40L and acute exacerbation frequency suggests asthma acute attacks may serve as a strong inflammatory trigger to upregulate OX40L expression, and high OX40L levels may elevate the risk of subsequent acute exacerbations, indicating a potential interactive association between the two.

This study's innovation resides in systematically investigating association patterns between OX40L and various immune-activation-related molecules from a serum biochemical characteristic perspective, defining its role in asthma by combining disease stratification and clinical indicators—filling the gap of insufficient systematic analysis in previous research. Meanwhile, through correlation and multiple linear regression analyses, this study first identifies IL-5 as the core independent factor associated with OX40L levels, providing direct clinical evidence for elucidating the potential interactive relationship between the two.

Even so, the present work has intrinsic shortcomings that call for improvement in subsequent studies. First, as a cross-sectional study, it only identifies correlations between OX40L and asthma status rather than confirming causal relationships between OX40L and disease progression. Future prospective cohort studies should dynamically track fluctuations in OX40L levels during patient therapy to further validate its association with disease outcomes. Second, the sample size is relatively limited, and all participants were recruited from a single institution, which may compromise the external validity of the findings. Subsequent multi-center, large-cohort studies should enroll asthmatic patients from different regions and ethnic groups to verify the general applicability of the conclusions. Third, this work failed to explore in depth the molecular mechanisms through which OX40L regulates asthma airway inflammation. Follow-up studies can utilize cell experiments or animal models, along with OX40L blocking antibodies or gene ablation approaches, to clarify the specific molecular targets of OX40L in asthma inflammation, laying a more precise theoretical basis for targeted therapies. Fourth, the association between OX40L levels and asthmatic patients' treatment response was not analyzed here. Future studies may include patients receiving standard therapy to explore whether OX40L can serve as a potential biomarker for assessing treatment outcomes.

From the clinical application perspective, OX40L boasts great clinical prospects as a promising therapeutic target. Currently, targeted agents against the OX40L-OX40 pathway have been tested in clinical trials for autoimmune disorders such as rheumatoid arthritis and psoriasis, achieving satisfactory therapeutic results. The findings of this study provide experimental evidence for the use of these agents in asthma treatment. Future research can further explore the therapeutic effect of anti-OX40L antibodies in asthmatic patients, especially those with moderate-to-severe asthma, thereby providing new therapeutic strategies. Meanwhile, as a disease-monitoring biomarker, OX40L can be combined with existing indicators to develop a more accurate asthma assessment model, guiding the formulation of personalized clinical treatment plans.

In summary, serum OX40L is abnormally upregulated in asthmatic patients and closely associated with immune activation-associated factors, lung function, and disease severity, making it a promising candidate for asthma assessment and targeted therapy. More basic and clinical studies are needed to further clarify its action mechanisms and clinical value, opening up new avenues for the precision diagnosis and treatment of asthma.

We further adjusted for potential confounding factors including medication use, asthma phenotypes, and allergy history in the multivariable regression model, and the core associations between OX40L and IL-5, lung function, and exacerbation frequency remained statistically significant, indicating the robustness of our findings.

## Conclusion

Serum OX40L, pro-inflammatory agents, chemokines and total IgE rise in asthmatics (with lower IL-10), increasing with severity and correlating positively with Th2 cytokines (notably IL-5) and negatively with FEV1%pred. IL-5, FEV1%pred and annual acute exacerbations independently regulate serum OX40L, which modulates asthma's immune-inflammatory network and serves as a potential severity biomarker for targeted therapies.

## Dodatak

### Conflict of interest statement

All the authors declare that they have no conflict of interest in this work.
